# Does malalignment affect patient reported outcomes following total knee arthroplasty: a systematic review of the literature

**DOI:** 10.1186/s40064-016-2790-4

**Published:** 2016-07-28

**Authors:** Mohammed Hadi, Tim Barlow, Imran Ahmed, Mark Dunbar, Peter McCulloch, Damian Griffin

**Affiliations:** 1 Trauma and Orthopaedic Department, UHCW, University of Warwick, Coventry, CV2 2DX UK; 2 John Radcliffe Hospital, University of Oxford, Oxford, OX3 9DU UK

## Abstract

**Background:**

Total knee replacement is an effective treatment for knee arthritis. While the majority of TKAs have demonstrated promising long-term results, up to 20 % of patients remain dissatisfied with the outcome of surgery at 1 year. Implant malalignment has been implicated as a contributing factor to less successful outcomes. Recent evidence has challenged the relationship between alignment and patient reported outcome measures. Given the number of procedures per year, clarity on this integral aspect of the procedure is necessary.

**Objective:**

To investigate the association between malalignment and PROMS following primary TKA.

**Methods:**

A systematic review of MEDLINE, CINHAL, and EMBASE was carried out to identify studies published from 2000 onwards. The study protocol including search strategy can be found on the PROSPERO database for systematic reviews.

**Results:**

From a total of 2107 citations, 18 studies fulfilled the inclusion criteria, comprising of 2214 patients. Overall 41 comparisons were made between a malalignment parameter and a PROM, with 30 comparisons (73 %) demonstrating no association. However, 50 % (n = 9) of the studies with ‘Low risk’ radiological assessment methods have reported a statistically significant association between one or more parameter of malalignment and PROMS.

**Conculsion:**

When considering malalignment in an individual parameter, there is an inconsistent relationship with PROMs scores. Malalignment may be related to worse PROMs scores, but if that relationship exists it is weak and of dubious clinical significance. However, this evidence is subject to limitations mainly related to the methods of assessing alignment post operatively and by the possibility that the premise of traditional mechanical alignment is erroneous. Larger longitudinal studies with a standardised, timely, and robust method for assessing alignment outcomes are required.

**Electronic supplementary material:**

The online version of this article (doi:10.1186/s40064-016-2790-4) contains supplementary material, which is available to authorized users.

## Background

Total knee Replacement (TKR) is considered an effective treatment for knee arthritis (Callahan et al. 1994). Over 77,000 TKA operations were performed during 2013 in England and Wales (Registry 2013) with expectations of increasing demand (Kane et al. 2003). While the majority of TKAs demonstrate significantly improved pain relief and function results (van Essen et al. 1998; March et al. 1999; Anderson et al. 1996), up to 20 % of patients remain unsatisfied with the outcome of surgery at 1 year (Kim et al. 2009; Scott et al. 2010; Baker et al. 2007; Robertsson et al. 2000; Bourne et al. 2010).

To ensure optimisation, an important technical objective during surgery is to achieve a perfect tri-planar component alignment (Sikorski 2008) with a neutrally aligned limb and a mechanical axis of 180° ± 3° and no tibio-femoral rotational mismatch (Ritter et al. 1994; Nicoll and Rowley 2010; Moreland 1988; Longstaff et al. 2009; Werner et al. 2005; Lotke and Ecker 1977; Bargren et al. 1983; Tew and Waugh 1985).

Three reasons to challenge the view that alignment in total knee replacements is of paramount importance have emerged (Eckhoff et al. 2005). Firstly, it is suggested that the evidence of poor outcomes secondary to malalignment is largely historic, based on studies of inferior implant designs (Bach et al. 2009; Bonner et al. 2011; Matziolis et al. 2010; Parratte et al. 2010), and the use of poor radiological techniques when assessing malalignment (Lotke and Ecker 1977). Secondly, outcomes following computer assisted TKA, proven to achieve better target alignment in comparison to conventional techniques, have demonstrated little evidence of clinical advantage (Matziolis et al. 2010; Cheng et al. 2012a). Thirdly, the choice of target for ideal alignment has been challenged by proponents of kinematically aligned TKA who have reported promising results (Howell et al. 2013a, b). Kinematic alignment aims to place the femoral component so that its transverse axis coincides with the primary transverse axis in the femur about which the tibia flexes and extends. As this axis is centred on the posterior condyles of the femur, which is not parallel to any standard coronal, sagittal or axial view, it is not measurable by standard means. With the removal of osteophytes the original ligament balance can be restored and the tibial component is placed with a longitudinal axis perpendicular to the transverse axis in the femur.

We performed a systematic review of the literature to answer the following research question: In patients undergoing primary total condylar knee replacement is malalignment, assessed radiologically, associated with functional outcomes and/or PROMs.

## Methods

This review followed the guidelines described by the Agency for Healthcare Research and Quality (AHRQ) criteria (Viswanathan et al. 2008a). The review has been registered and published on the PROSPERO database; Protocol Number 2012:CRD42012001914 (Hadi et al. 2012).

### Literature search

A literature search of the following databases was carried out: Medical Literature Analysis and Retrieval System Online, Bethesda, Maryland, USA (MEDLINE), Cumulative Index to Nursing and Allied Health Literature, Glendale, California USA (CINHAL), Excerpta Medica Database, Amsterdam, the Netherlands (EMBASE). A broad search strategy using MeSH terms “knee”, “replacement”, “alignment” and “outcome” was adopted. This was intended to identify English-language studies published from 2000 through to 2014. The search was restricted to this period to avoid the inclusion of studies with potentially poor implant designs and weak radiological assessment methods. The search was last performed on September 2014.

### Eligibility criteria

Both observational and experimental designs were considered for inclusion in this review.

Inclusion criteria:

- All patients who were deemed eligible for a primary TKA were considered.
- All open procedures that used a total condylar knee replacement.
- All described approaches.
- All radiological alignment assessment methods and parameters described.

Exclusion criteria:

- Studies that have fulfilled the inclusion criteria but have not provided adequate or clear information on the correlation analysis between malalignment and PROMs.
- Studies with a mean follow-up of <6 months,
- Abstract-only publications, expert opinions and chapters from books.

### Extraction of data

Two investigators (MH, TB) independently reviewed the titles and abstracts to identify and retrieve all articles relevant to our research questions, disagreements were settled by consensus between the two reviewers or with a third investigator (MD).

**Fig. 1:**
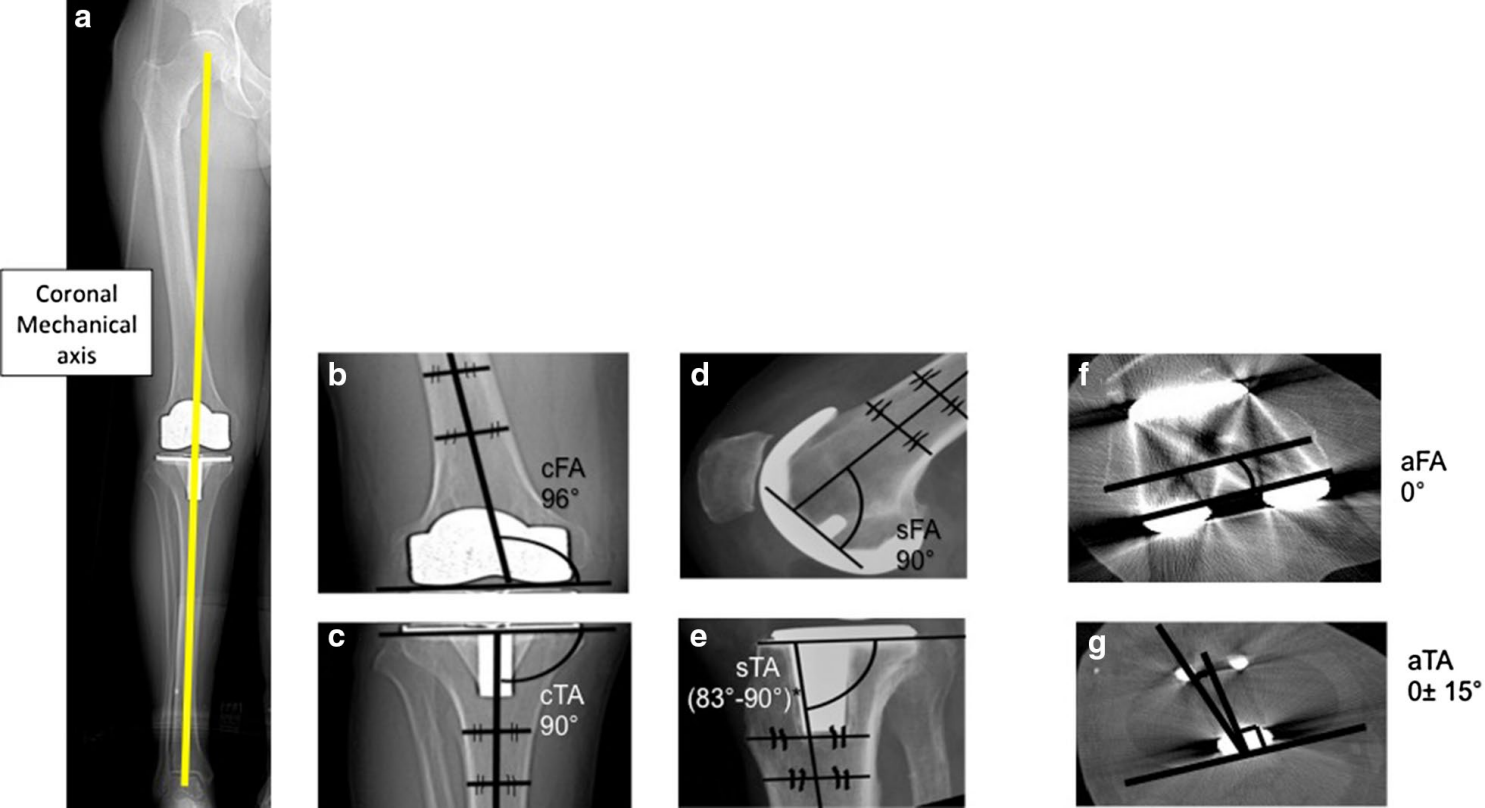
A diagrammatic representation of different alignment parameters based on The Knee Society Total Knee Arthroplasty Roentgenographic Evaluation and Scoring System (Viswanathan et al. 2008a). The Coronal Tibiofemoral mechanical angle is the angle resulting from drawing a line from the centre of the femoral head down to centre of the ankle through the centre of the knee **a** ideally 180°. The coronal femoral angle cFA **b** ideally 96°—and coronal tibial angle cTA, **c** ideally 90°—are the angles between the components’ coronal axes (the line connecting the femoral components most distal condyles and the line along the horizontal tibial plate) and the bones’ coronal anatoical axes (line which bisects the medullary canal of the femur and tibia respectively). The coronal tibiofemoral anatomical angle is a combination of the coronal anatomical femoral axis and coronal anatomical tibial axis. The sagittal femoral sFA, **d** ideally 90°—and sagittal tibial sTA, **e** ideally between 83° and 90°—angles are the angles between the components’ sagittal axes (*horizontal line* perpendicular to the femoral component peg and line along the horizontal tibial plate) and the anatomical sagittal bones’ axes (line which bisects the medullary canal of the femur and tibia respectively). The axial femoral (aFA) **f** ideally 0°—and axial tibial—ideally within 15°—(aTA), **g** angles are the angles between the components’ axial axes (line through the centre of the femoral pegs and the line through the most posterior points of the tibial plate on axial views respectively) and the bones’ axial axes (surgical epicondylar femoral axis and the tibial tuberosity axis respectively). The combined components axial (aCRA) rotational alignment angles—ideally 0°—is the angle between the components axial axes

The parameters of malalignment are illustrated in Fig. 1. For the purposes of this review we describe coronal alignment as the mechanical alignment, and describe the method of assessment (long leg or short leg radiograph).

### Quality assessments of included studies

All studies were assessed for their methodological qualities in accordance with their study design. Case control and Cohort studies were assessed using the Ottawa–Newcastle score system (Stang 2010). RCTs and Case series were assessed using an AHRQ design-specific scales (Viswanathan et al. 2008b).

Studies were further evaluated based on the quality of their radiological methods for assessing alignment. The evaluation was done using a five-question checklist devised for this review; the Radiological Assessment Quality (RAQ) criteria (Hadi et al. 2015). The items in the checklist together with their corresponding justification are described in the Additional file 1. Studies were deemed as low, unclear or high risk of assessment bias based on the radiological methods described. Sensitivity analysis using radiological assessment quality did not alter the results.

### Statistical analysis

Due the exploratory nature of the research question, the summary of data was focused on descriptive statistics and qualitative assessment of the content of the identified literature. Meta-analysis was not part of the study protocol and was not conducted as the outcome measures, measure of alignment, and methods of assessment were heterogeneous. Given these limitations, it was thought it would produce a precise, but potentially spurious result (Egger et al. 1998).

## Results

### Search results

**Fig. 2:**
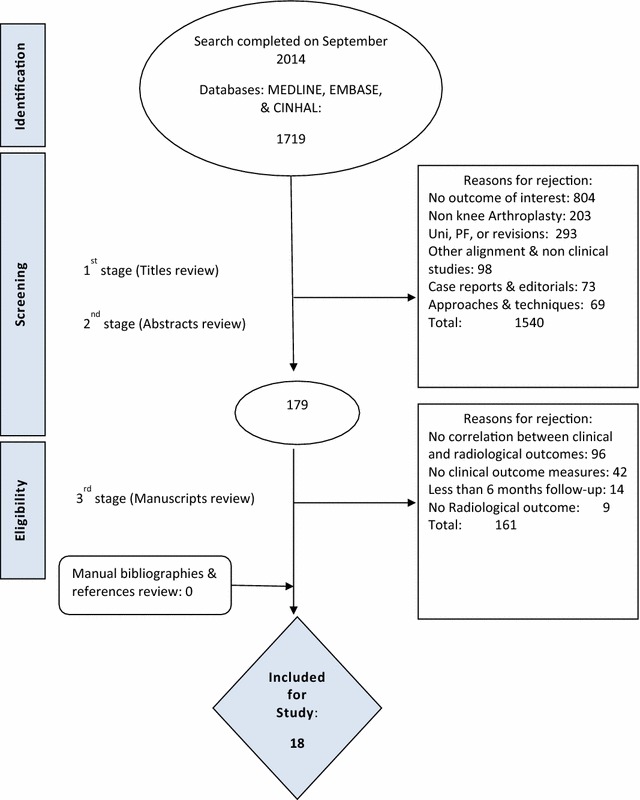
PRISMA flow diagram including the details of our search results for this review. Figure shows the reasons behind study exclusion at each stage of the search and the number of studies identified at each point of the search

The initial search returned 2107 citations, of which 1719 were considered for screening. Details of the study selection process are described in Fig. 2.

A total of 18 studies (Nicoll and Rowley 2010; Longstaff et al. 2009; Bach et al. 2009; Matziolis et al. 2010; Howell et al. 2013b; Aglietti et al. 2007; Bankes et al. 2003; Barrack et al. 2001; Bell et al. 2014; Blakeney et al. 2014; Choong et al. 2009; Czurda et al. 2010; Gothesen et al. 2014; Huang et al. 2012; Lutzner et al. 2010; Magnussen et al. 2011; Rienmuller et al. 2012; Stulberg et al. 2008) fulfilled the review inclusion criteria, including five RCTs (Blakeney et al. 2014; Choong et al. 2009; Gothesen et al. 2014; Huang et al. 2012; Lutzner et al. 2010), seven case control studies (Nicoll and Rowley 2010; Matziolis et al. 2010; Barrack et al. 2001; Bell et al. 2014; Czurda et al. 2010; Magnussen et al. 2011; Stulberg et al. 2008) and 6 case series (Longstaff et al. 2009; Bach et al. 2009; Howell et al. 2013b; Aglietti et al. 2007; Bankes et al. 2003; Rienmuller et al. 2012). The methodological quality-assessment of included studies is presented in Tables 1, 2 and 3. The results did not alter with subgroup analysis based on quality assessment.

**Table 1:** Quality assessment criteria for RCTs

Authors	Quality assessment									Judgment on risk of bias
	Was the allocation sequence generated adequately?	Was the allocation of treatment adequately concealed	Did researchers rule out any unintended exposure that might bias results?	Were participants analysed within the groups they were originally assigned to?	Was the length of follow-up different between the groups	Were the outcome assessors blinded to the intervention or exposure status of participants?	Were the potential outcomes pre-specified by the researchers? Are all pre-specified outcomes reported?	If attrition was a concern were missing data handled appropriately?	Were outcomes assessed using valid and reliable measures across all study participants?	
Blakeney et al. (2014)	Yes	No	Yes	Yes	No	No	Yes	Yes	Yes	Low risk
Choong et al. (2012)	Yes	No	Yes	Yes	No	No	Yes	Yes	Yes	Low risk
Huang et al. (2012)	Yes	No	Yes	Yes	No	No	Yes	Yes	Yes	Low risk
Lutzner et al. (2010)	Yes	No	Yes	Yes	No	No	Yes	Yes	Yes	Low risk
Gothesen et al. (2014)	Yes	Yes	Yes	Yes	No	Yes	Yes	Yes	Yes	Low risk

Assessed using AHRQ design specific scale (Stang 2010)

**Table 2:** Quality assessment of Case control and Cohort studies

Author	Quality assessment of case control studies								
	Is the case definition adequate?	Representativeness of the cases	Selection of controls	Definition of controls	Comparability of cases and controls on basis of design or analysis	Ascertainment of exposure	Same method of ascertainment for cases and controls	Non-Response rate	Total Newcastle Ottawa Scale (possible 9 starts)
Barrack et al. (2001)	Yes	Yes	Yes	Yes	Yes	Yes	Yes	Yes	8*
Bell et al. (2014)	Yes	Yes	Yes	Yes	Yes	Yes	Yes	Yes	8*
Czurda et al. (2010)	Yes	Yes	Yes	Yes	Yes	No	Yes	Yes	7*
Magnussen et al. (2011)	Yes	Yes	Yes	Yes	Yes	No	Yes	Yes	7*
Matziolis et al. (2010)	Yes	Yes	Yes	Yes	Yes	Yes	Yes	Yes	8*
Nicoll and Rowley (2010)	Yes	Yes	Yes	Yes	Yes	Yes	Yes	Yes	8*
Stulberg et al. (2008)	Yes	Yes	Yes	Yes	Yes	Yes	Yes	Yes	8*

Assessed using the Ottawa-Newcastle score (Viswanathan et al. 2008a)

* Represents how many stars were achieved in the assessment of quality for each study

**Table 3:** Quality assessment of Case series studies

Author	Quality assessment of case series			
	Consecutive selection of patients?	Were outcomes measured in an objective way?	Were confounders identified and controlled?	Was follow up sufficiently long and complete
Aglietti et al. (2007)	Yes	?	No	Yes
Bach et al. (2009)	Yes	?	No	Yes
Bankes et al. (2003)	Yes	Yes	No	Yes
Howell et al. (2013b)	?	Yes	Yes	Yes
Longstaff et al. (2009)	Yes	Yes	Yes	Yes
Rienmüller et al. (2012)	Yes	?	No	Yes

Assessed using AHRQ design specific scale (Stang 2010)

The total number of patients recruited in all included studies was 2214 patients. Minimal patient baseline characteristics were reported, however, where reported they were comparable between studies. Study characteristics can be seen in Table 4.

**Table 4:** Study characteristics of included studies in this review

Author and journal	Study design	Sample size	Follow up (mean range)	Number of patients lost to follow up	Final study sample size
Choong et al. (2012) J Athro	RCT (single centre)	120	1 year	9	111
Lutzner et al. (2010) Knee Surg. Sports Trauma Arthos	RCT (single centre)	80	1.8 years	7	73
Huang et al. (2012) Journal of Arthoplasty	RCT (single centre)	111	5 years	21	90
Blakeney et al. (2014) The Knee	RCT (single centre)	107	46 months	14	93
Gothesen et al. (2014) JBJS	RCT (multi-centre)	194	5 years	19	175
Barrack et al. (2001) CORR	Case control (single centre)	30	5.7 years	2	28
Stulberg et al. (2008) Orthopaedics	Case control (single centre)	58	2.5 years	6	52
Nicoll and Rowley (2010) JBJS	Case control (single centre)	61	>1 year	23	39
Matziolis et al. (2010) Arch Orthop Trauma Surg	Case control (single centre)	218 (from a database)	5–10 years	168	50
Czurda et al. (2010) Knee Surg Sport Trau Arthrosc	Case control (single centre)	38	2.2 years	0	38
Magnussen et al. (2011) CORR	Case control (single centre)	608	Median 4.7 years (2–19.8)	55	553
Bell et al. (2014) The Knee	Case control (single centre)	127	1 year	15	112
Bankes et al. (2003) The Knee	Case series (single centre)	198	6.5 years	0	198
Aglietti et al. (2007) CORR	Case series (single centre)	64	8 Years	19	53
Longstaff et al. (2009) J Arthro	Case series (single centre)	159	1 year	9	146
Bach et al. (2009) The Knee	Case series (single centre)	105	10.8 years	7	98
Rienmüller et al. (2012) International Orthopaedics	Case series (single centre)	219	5 Years	15	204
Howell et al. (2013b) Knee Surg Sport Trau Arthrosc	Case series (single centre)	101	6–9 months	1	101

The functional and PROMS outcomes identified in this review included: Knee Society Score (KSS), Hospital for Special Surgery Score (HSS), Western Ontario and McMaster Universities Osteoarthritis Index (WOMAC), SF-12, SF-36, EuroQol, patella-femoral symptoms Score, Bristol score, Nottingham health profile, Visual analogue scale (VAS).

Out of the possible malalignment parameters considering the component’s six degrees of freedom, ten parameters were reported. Multiple different measures were used to measure coronal alignment, with varying nomenclature. Sagittal and axial alignments of the tibial and femoral components were not subject to this confusing nomenclature. See the Additional file 1 for a full description of each alignment parameter with detailed findings from each paper.

### Quality assessment

Tables 1 and 2 demonstrate the quality assessment of each included study.

### Radiological assessment

Table 5 demonstrates the radiological characteristics of each study using the RAQ criteria.

**Table 5:** Radiological methods quality assessment of included studies

Author	Modality of image	Timing of image	Weight bearing	Protocol/standardisation	Rater reliability assessment	Outcome
Choong et al. (2012)	CT, LLR	6 weeks	Y	Y	N	Low risk
Lutzner et al. (2010)	CT, LLR	18–32 months	Y	U	N	High risk
Huang et al. (2012)	CT, LLR	6 weeks	Y	Y	N	Low risk
Blakeney et al. (2014)	CT (3D)	3 months	N	Y	N	Medium risk
Gothesen et al. (2014)	CT, LLR	3 months	Y	Y	N	Low risk
Barrack et al. (2001)	CT, LLR	At latest follow up	Y	U	N	High risk
Stulberg et al. (2008)	LLR, SLR, Navigation system	4 weeks and 2 years	Y	Y	N	Low risk
Nicoll and Rowley (2010) JBJS	CT, SLR	At least 1 year after TKR	N	U	N Senior author	High risk
Matziolis et al. (2010)	LLR	Latest follow up	Y	Y	Y	High risk
Czurda et al. (2010)	CT, LLR	At 1st follow up	Y	Y	N Independent radiologist	Low risk
Magnussen et al. (2011)	LLR	Follow up (varied)	Y	Y	Y	High risk
Bell et al. (2014)	CT	26 months	N	U	MSK radiologist	High risk
Bankes et al. (2003)	SLR	3 and 12 month follow up	Y	Y	N	Low risk
Aglietti et al. (2007)	LLR	Latest follow up	Y	Stress to assess varus-valgus stability	N	High risk
Longstaff et al. (2009)	CT	6 months	N	Y	Y	Low risk
Bach et al. (2009)	SLR	At follow up	N	Y	N Experienced radiologist	High risk
Rienmüller et al. (2012)	LLR, Axial XR	5 years	N	Y	Y	High risk
Howell et al. (2013b)	CT	2 days	N	Y	N	Medium risk

We devised a 5 point checklist (Fig. 2) and all studies were assessed using this checklist to identify whether they were high/low risk

*CT* computerised tomography, *LLR* long leg radiograph, *SLR* short leg radiograph, *Y* yes, *N* no, *U* unknown

### Association between malalignment and patient reported outcome measures (PROMs)

Overall 41 comparisons were made between a malalignment parameter and a PROM, with 30 comparisons (73 %) demonstrating no association. Of the 18 studies, 12 studies (67 %) demonstrated an association between malalignment in one or more parameter of alignment and a worse patient reported outcome. Of these, nine studies (50 %) applied radiological methods with a low or medium risk of bias.

We summarised the association between malalignment and PROMs according to the plane of assessment and the individual components.

**Fig. 3:**
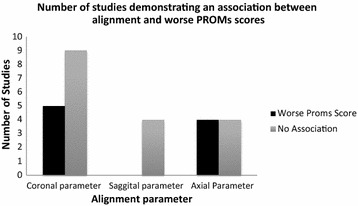
Graph demonstrating the number of studies demonstrating an association between malalignment and worse PROMs (patient reported outcome measures) scores based on imaging in the coronal, sagittal and axial view

In the coronal plane, five out (Longstaff et al. 2009; Cheng et al. 2012a; Aglietti et al. 2007; Blakeney et al. 2014; Huang et al. 2012) of fourteen studies (Nicoll and Rowley 2010; Longstaff et al. 2009; Bach et al. 2009; Matziolis et al. 2010; Cheng et al. 2012a; Howell et al. 2013b; Aglietti et al. 2007; Bankes et al. 2003; Blakeney et al. 2014; Czurda et al. 2010; Gothesen et al. 2014; Huang et al. 2012; Magnussen et al. 2011; Stulberg et al. 2008) showed an association between malalignment in the coronal plane and worse PROM scores (Table 6; Fig. 3) provide graphical representation of this.

**Table 6:** Association between coronal malalignment and worse outcome

Author	Sample size	Type of radiograph	RAQ score	Outcome measure	Malalignment parameter	Association between malalignment and worse outcome
Aglietti et al. (2007)	53	LL	High risk	KSS (Clinical) HHS Patella score	cTFmA	Yes
Choong et al. (2012)	111	LL	Low risk	IKS SF-12	cTFmA	Yes
Blakeney et al. (2014)	93	CT	Medium risk	SF-12 OKS	cTFmA	Yes
Huang et al. (2012)	90	LL	Low risk	IKS SF-12	cTFmA	Yes
Longstaff et al. (2009)	146	CT	Low risk	KSS	cTA, cFA	Yes
Howell et al. (2013b)	101	CT	Medium risk	OKS WOMAC	cTFmA, cTA	No
Magnussen et al. (2011)	553	LL	High risk	KSS	cTFmA, cTA, cFA	No
Matziolis et al. (2010)	50	LL	High risk	KSS WOMAC SF36KSS	cTFmA, cTA, cFA	No
Stulberg et al. (2008)	52	LL	Low risk	KSS	cTFmA	No
Gothesen et al. (2014)	175	LL	Low risk	KSS	cTFmA, cTA, cFA	No
Czurda et al. (2010)	38	LL	Low risk	WOMAC KSS	cTFmA, cFA	No
Bach et al. (2009)	98	SL	High risk	KSS, HSS, Bristol score, NHP	cTFaA, cTA, cFA	No
Bankes et al. (2003)	198	SL	Low risk	KSS	cTFaA, cTA, cFA	No
Nicoll and Rowley (2010)	45	SL	High risk	KSS	cTFaA, cTA, cFA	No

*KSS* knee society score, *HHS* harris hip score, *NHP* Nottingham health profile, *WOMAC* Western Ontario and McMaster Universities Arthritis index, *OKS* Oxford knee score, *SF*-*12* short form-12, *cTFmA* coronal tibio-femoral mechanical alignment, *cTFaA* coronal tibio-femoral anatomical alignment, *cTA* coronal tibial alignment, *cFA* coronal femoral alignment, *LL* long leg radiograph, *SL* straight leg radiograph

Only four studies (Longstaff et al. 2009; Bach et al. 2009; Bankes et al. 2003; Stulberg et al. 2008) investigated sagittal malalignment and its relationship with PROM, with none of these demonstrating a statistically significant association between femoral or tibial malalignment and worse outcomes (Table 7; Fig. 3).

**Table 7:** Association between sagittal malalignment and worse outcome

Author	Sample size	Type of radiograph	RAQ score	Outcome measure	Malalignment parameter	Association between malalignment and worse outcome
Bankes et al. (2003)	198	SL	Low risk	KSS	sFA, sTA	No
Bach et al. (2009)	98	SL	High risk	KSS, HSS, Bristol score, NHP	sFA, sTA	No
Stulberg et al. (2008)	52	LL	Low risk	KSS	sFA, sTA	No
Longstaff et al. (2009)	146	CT	Low risk	KSS	sFA, sTA	No

*KSS* knee society score, *HSS* hospital for special surgery score, *NHP* Nottingham health profile, *sTA* sagittal tibial angle, *sFA* sagittal femoral angle, *LL* long leg radiograph, *SL* straight leg radiograph

Four (Barrack et al. 2001; Bell et al. 2014; Czurda et al. 2010; Lutzner et al. 2010) out of eight studies (Nicoll and Rowley 2010; Longstaff et al. 2009; Howell et al. 2013b; Barrack et al. 2001; Bell et al. 2014; Czurda et al. 2010; Lutzner et al. 2010; Rienmuller et al. 2012) found a statistically significant association between malalignment in the axial view and worsening patient reported outcome measures (Table 8; Fig. 3).

**Table 8:** Association between axial malalignment and worse outcome

Author	Sample size	Type of radiograph	RAQ score	Outcome measure	Association between malalignment and worse outcome
Barrack et al. (2001)	28	CT, LLR	High risk	KSS	Yes
Bell et al. (2014)	112	CT	High risk	OKS VAS	Yes
Lutzner et al. (2010)	73	CT, LLR	High risk	KSS	Yes
Czurda et al. (2010)	38	CT, LLR	Low risk	WOMAC KSS	Yes
Rienmüller et al. (2012)	204	LLR, Axial XR	High risk	KSS	No
Howell et al. (2013b)	101	CT	Medium risk	OKS WOMAC	No
Nicoll and Rowley (2010)	45	CT, SLR	High risk	KSS	No
Longstaff et al. (2009)	146	CT	Low risk	KSS	No

*KSS* knee society score, *WOMAC* Western Ontario and McMaster Universities Arthritis Index, *OKS* Oxford knee score, *VAS* visual analogue score for pain, *LL* long leg radiograph, *SL* straight leg radiograph

**Fig. 4:**
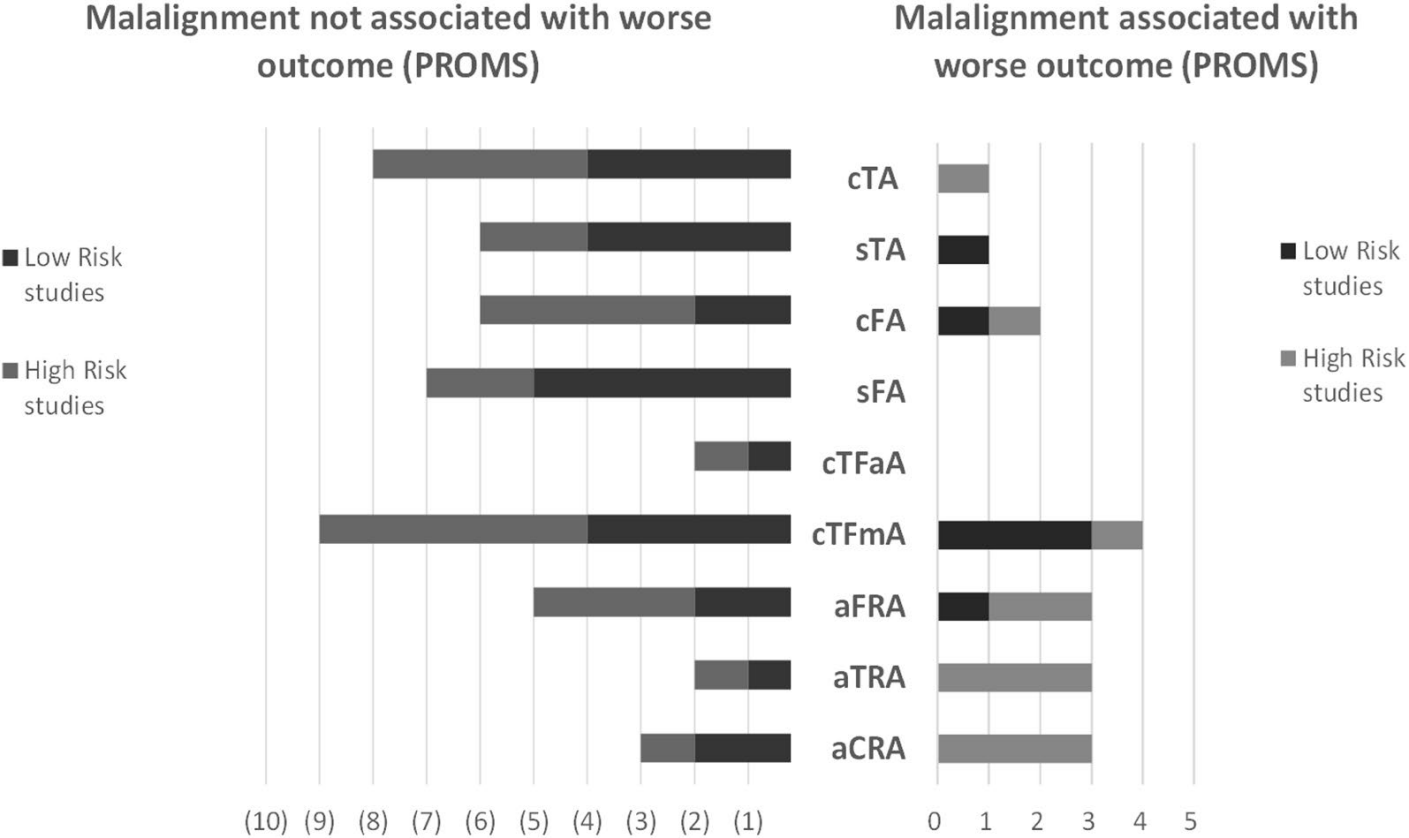
Graph demonstrating rate each malalignment parameter is reported to associate with outcome. Studies are divided into with low and high risk of radiological assessment bias as per he RAQ criteria. *cTA* coronal tibial angle, *sTA* sagittal tibial angle, *cFA* coronal femoral angle, *sFA* sagittal femoral angle, *cTFmA* coronal tibio-femoral mechanical angle, *cTFaA* coronal tibiofemoral anatomical angle, *aFRA* axial femoral rotational angle, *aTRA* axial tibial rotational angle, *aCRA* axial combined rotational/mismatch angle

Finally, Fig. 4 demonstrate the rate each individual malalignment parameter’s association with PROMS outcome. The chart highlights the number of studies with low and high risk for radiological assessment bias as per the RAQ criteria.

## Discussion

The main findings of this review were that 50 % (n = 9) of the studies with ‘Low risk’ radiological assessment methods have reported a statistically significant association between one or more parameter of malalignment and PROMS. Overall 41 comparisons were made between a malalignment parameter and outcome within the included studies, with only 11 comparisons (27 %) demonstrating an association. With a *p* value of 0.05, we would expect two of these associations by chance. This suggests that the effect of malalignment on PROMs is likely to be small and it is unclear from this review the clinical significance of this finding. When assessing each parameter individually:

### Coronal malalignment

In the literature, coronal malalignment is seen regarded as one of the most important factors determining the long-term prosthesis survival. Several authors stressed the importance of restoring limb coronal mechanical alignment to within 180°. In this review, as many as 64 % of studies investigating alignment in the coronal plane showed no associated between malalignment and worse outcome measures.

### Sagittal malalignment

Components malalignment on this plane can alter the posterior tibial slop and affect the flexion and extension gaps. This may result in overstuffing and limited joint range. Femoral notching can be seen in excessive femoral component extension position. However, 100 % of studies reviewed in this review showed no associated between sagittal malalignment and worse outcome measures.

### Axial malalignment

Many references exist for measuring femoral and tibia component rotation. Individual component malalignment and combined mismatch can result in abnormal patella tracking and subsequent anterior knee pain. Our review show 50 % of studies found an association between malalignment and worse PROMs.

### Strengths and limitations

Several caveats exist in interpreting this paper, mostly based on the limitations of the studies involved, and the complexity of the topic in general. There is a lack of consistency in the way different studies assessed alignment following a TKA. For example, the use of long leg and short leg radiographs. When using long leg radiographs a direct comparison between the mechanical axis and the femoral and tibial alignment can be made in the coronal plane. If using a short leg radiograph an indirect assessment is made, based on the assumption that the intramedullary canal of the femur deviates 6° from the mechanical axis. In reality this deviation is variable, making this assessment method less accurate. To address this, a RAQ checklist was devised to assess the radiological methods, although this did not alter the overall results of the review by sub-analysis. These variations in methodology (combined with variation in PROMS scores) make meta-analysis problematic.

Furthermore, a number of studies restricted their analysis to one or two parameters of alignment. This approach is problematic given the relative interconnection between the alignment components in a TKA (Berend et al. 2004; Ritter et al. 2011). Berend et al. (2004) found the effect of malalignment in one implant moderated by the alignment of the other. Ritter et al. (2011) concluded that correction of the alignment of the second component in order to produce an overall neutrally aligned knee replacement when the first component has been malaligned may increase the risk of failure. These findings suggest a complex interplay between all measures of alignment in both the tibial and the femoral components that cannot be simplified to conventional definitions of “malaligned” or “aligned”. Given that some studies did not report findings for certain parameters there is the potential for publication bias as studies where no relationship was found contained missing data.

In addition, the parameters of malalignment were poorly defined. Studies presented malalignment data either in terms of deviation from the leg axis in the arithmetic mean or as two groups of ‘Aligned’ versus’ Malaligned’ or ‘Outliers’. While the majority of studies applied a ±3° range around a perfect alignment measurement, some studies had a more stringent criterion applying a ±2° range. Applying this narrow range, Longstaff et al. (2009) found better functional outcomes with good coronal femoral alignment and only a trend to better function at 1 year on patients with ‘good’ coronal tibial and ‘good’ sagittal tibial and sagittal femoral alignment.

The characteristics of the patient-related clinical outcome measures used by the studies included in this review may have contributed to the quality of the evidence presented. Some quality of life outcomes can suffer from ceiling effects that can result in abolishing the advantage of perfect aligned implants in comparison to those with mild degree of malalignment. The KSS, which is a regularly used functional score and most commonly identified in this review is subject to assessor bias.

Seven studies in this review used CAS. This is relatively small given the popularity of this technique and its consistency at achieving better alignment (Cheng et al. 2012b; Fu et al. 2012; Hetaimish et al. 2012). It would be reasonable to assume that studies reporting CAS outcomes would provide data on the association between alignment and outcome. However, the literature suggest that CAS surgery studies are usually under powered for sub-analysis of aligned versus malaligned and therefore not reported (Khan et al. 2012). Eckhoff et al. assessed CT scans on 90 patients to investigate axial limb alignment. They found that normal individuals expressed a wide range in the straight-line mechanical axis. This has two consequences; if surgical correction of a pathological knee to achieve a straight mechanical axis does not return the mechanical alignment to normal. This can lead to increased pressures on the polyethylene components increasing wear rates. Secondly, if a knee is not straight, the procedures achieve mechanical alignment will alter soft tissue balance affecting PROMs scores. This study has important implications for CAS surgery, if the algorithms do not incorporate this wide variation in natural morphology and kinematics of the knee (as evidenced by Eckhoff) the end result of CAS surgery can lead to further malaligned knees, increased wear and worsening PROMs scores (Eckhoff et al. 2005).

When our results are viewed from the kinematic perspective the unclear association between mechanical alignment and outcome makes sense given that there is a large variation in the anatomy of femora and tibiae and that most patients do not have a neutral hip–knee–ankle axis (Hollister et al. 1993). It is entirely possible that an anatomically (kinematically) aligned, but mechanically malaligned, implanted prosthesis could recreate a patient’s preoperative kinematics. Howell et al. (2015) concluded that kinematic aligned knee replacement did not adversely affect implant survival or function as it restores the constitutional alignment of the limb and joint line, subsequently avoiding collateral ligament imbalances. This would create a group of patients that, for the purposes of the studies included in this review, were “malaligned”, but had good PROMs scores based on their alignment. This could explain the dubious relationship demonstrated between alignment and outcome.

In conclusion, alignment in an individual parameter may have a weak, and perhaps clinically insignificant, effect on scores. However, this evidence is subject to limitations mainly related to the methods of assessing alignment post operatively. Larger longitudinal studies with a standardised, timely, and robust method for assessing alignment outcomes are required.

## Additional file

10.1186/s40064-016-2790-4 Assessment tool used to assess the radiological criteria used in each study.
