# Supplementary material for: Does malalignment affect patient reported outcomes following total knee arthroplasty: a systematic review of the literature
Source: Springerplus. 2016 Jul 28;5(1):1201. doi: 10.1186/s40064-016-2790-4 (PMC4963339; doi:10.1186/s40064-016-2790-4)
Supplement: Supplementary file 1 — 10.1186/s40064-016-2790-4 Assessment tool used to assess the radiological criteria used in each study. [file 40064_2016_2790_MOESM1_ESM.docx]

| Table 1-s: Table summarising the results of the studies investigating the association between malalignment in the cTFmA/cTFaA and patient reported outcome measures.  cTFmA. Studies investigating the association between coronal Tibio-femoral mechanical angle (cTFmA) malalignment and revision rates. | | | | | |
| --- | --- | --- | --- | --- | --- |
| Author | Outcome Measure | Association between malalignment and worse outcome | Sample size | Alignment data | Findings |
| Aglietti et al 2007 | KSS (Clinical)  HHS (Harris hip score) Patella score | Yes | 53 | 69% within 2° of neutral alignment. 19% varus within 5°. 11% Valgus within 5°. 2 knees (4%) had a varus alignment greater than 5°. | Significant Lower functional scores in patients with overall varus alignment. (p=0.001) |
| Choong et al 2009 | IKS  SF-12 | Yes | 111 | 88% in the computer assisted group had alignment within 3°. 61% in the conventional group had alignment within 3°. | The total IKS score was significantly better in patients with a mechanical axis within 3° of neutral compared to those greater than 3° (p,0.001). The SF-12 physical scores were also significantly better for patients with a mechanical axis within 3° of neutral (p=0.03 at 6 months). |
| Blakeney et al 2013 | SF-12  OKS | Yes | 93 | 74 participants were within 3° of alignment and 32 were >3° of alignment. | There was a significant improvement in the OKS when mechanical axis was within ±3° of neutral: p = 0.045. There were no statistically signiﬁcant differences seen in the MCS and PCS components of the SF-12. |
| Huang et al 2012 | IKS  SF-12 | Yes | 90 | 69 participants were within 3° of alignment and 21 were >3° of alignment. | IKS score signiﬁcantly better for patients with a mechanical axis within 3° at 5 years 142 [123-179] vs 129 [80-157]) (P = .028).. |
| Howell et al 2013 | OKS  WOMAC | No | 101 | 93 knees within 0±3°, 6 were >3° varus and 1 was >3° valgus. | No significant difference in OKS and WOMAC scores between the groups. |
| Magnussen et al 2011 | KSS | No | 553 | 181 knees classed as residual varus (<177°) and 352 classed as neutral (177° - 183°) | No significant difference in KSS scores between the two groups (p=0.12) |
| Matziolis et al 2010 | KSS  WOMAC  SF36 | No | 50 | Deviation of mechanical axis from arithmetic mean:  Group A (25 participants): 6.3°±2°  Group B (25 participants): 0°±1.2° | No significant difference in any of the PROMs scores assessed between the two groups. |
| Stulberg et al 2008 | KSS | No | 52 | The mean cTFmA for the 52 patients was  0.56° ± 1.0° (-1-3) of valgus | No significant difference between malalignment and KSS scores |
| Gothesen et al 2014 | KSS | No | 175 | Data not available | No significant difference between malalignment between groups and KSS scores |
| Czurda et al 2010 | WOMAC  KSS | No | 38 | No data provided | No significant difference in PROMs scores between the two groups |
| cTFaA. Studies investigating the association between coronal Tibio-femoral anatomical angle (cTFaA) malalignment and revision rates. | | | | | |
| Author | Outcome Measure | Association between malalignment and worse outcome | Sample size | Alignment data | Findings |
| Bach et al 2009 | KSS, HSS, Bristol score, Nottingham health profile | No | 98 | 48 participants (48.9%) were within normal range. | No significant correlation was found between the prosthetic alignment (femorotibial angle) and the mean clinical score outcome (p>0.05). |
| Bankes et al 2003 | KSS | No | 198 | 4-10° of valgus was achieved in 64.6% of patients, <4° in 31.4% and >10° in 4% | There was no significant difference between knees in acceptable and suboptimal alignment in terms postoperative knee function scores. |
| Nicol et al 2010 | KSS | No | 45 | 13 outliers classed as >6±3° valgus | There was no significant difference between malalignment in this parameter and KSS score |

**cTFmA -** coronal Tibio-femoral mechanical angle, **cTFaA -** coronal Tibio-femoral anatomical angle, **KSS – Knee society score, WOMAC - Western Ontario and McMaster Universities Arthritis Index, OKS – Oxford knee score, SF-12 – Short form – 12.**

| Table 2-s: Table summarising the results of studies investigating the association between alignment in both the cTA and CFA and patient reported outcome measures | | | | | | | |  |  |  |
| --- | --- | --- | --- | --- | --- | --- | --- | --- | --- | --- |
| Author | | Outcome Measure | Association between malalignment and worse outcome | Sample size | Alignment data | Findings | |  |  |  |
| Bach et al 2009 | | KSS, HSS, Bristol score | No | **98** | 93.8% participants within normal range - mean tibial angle of 86.8°. | No significant correlation was found between the prosthetic alignment (coronal tibial angle) and the mean clinical score outcome (p>0.05). | |  |  |  |
| Bankes et al 2003 | | **KSS** | **No** | **198** | Mean coronal tibial component angle was 88.2°, with 19.2% <87° and 0.5% >93° | No significant difference between knees in acceptable and suboptimal alignment in terms postoperative knee function scores. | |  |  |  |
| Howell et al 2013 | | OKS  WOMAC | No | 101 | 96 knees varus outliers (>0°) and 6 knees were ≤0°. | No significant difference in WOMAC and OKS scores. | |  |  |  |
| Magnussen et al 2011 | | KSS | Yes | 553 | Postoperative cTA was varus in 35 knees (<87°), neutral in 514 (87°-93°), and valgus in 4 knees (>93°) | Statistically signicant lower IKS scores when the tibial component was in varus compared with neutral alignment (p = 0.002). | |  |  |  |
| Matziolis et al 2010 | | KSS  WOMAC  SF36 | No | 50 | Absolute malpositioning of the tibial component:  Group A (25 participants): 1.8°±1.9°  Group B (25 participants): -0.3°±1.4° | No significant difference in any of the PROMs scores assessed between the two groups. | |  |  |  |
| Gothesen et al 2014 | | **KSS** | **No** | **175** | No data provided | Patients with a tibial posterior slope < 1°, or an anterior slope, had worse KSS scores and worse KOOS subscale scores for quality of life at three months. | |  |  |  |
| Nicol et al 201 | | **KSS** | **No** | **45** | 9 outliers classed as >0±3° | There was no significant difference between malalignment in this parameter and KSS score | |  |  |  |
| Longstaff et al 2009 | | KSS | No | **146** | 122 knees classed as good (-2° - +2° of neutral). 24 classed as bad (outside the ‘good’ parameters) | Patients with a good coronal tibial alignment demonstrated a trend to better function at 1 year. However this relationship was non-significant | |  |  |  |
| cFA- Studies investigating the association between coronal femoral angle (cFA) malalignment and Patient reported outcome measures | | | | | | | |  |  |  |
| Author | Outcome Measure | | Association between malalignment and worse outcome | Sample size | Alignment data | | Findings |  |  |  |
| Bach et al 2009 | KSS, HSS, Bristol score | | No | **98** | 100% of participants within normal range with a mean femoral angle of 96.2°. | No significant correlation was found between the prosthetic alignment (coronal femora angle) and the mean clinical score outcome (p>0.05). | |  |  |  |
| Bankes et al 2003 | **KSS** | | **No** | **198** | Mean coronal femoral component angle was 96.05° with 11.6% having values < 948° and 0.5% >100° | No significant difference between knees in acceptable and suboptimal alignment in terms post operative knee function scores. | |  |  |  |
| Magnussen et al 2011 | KSS | | Yes | 553 | **Postoperative cFA was varus in 24 knees** (<87°)**, neutral in 513** (87°-93°)**, and valgus in 16** (>93°). | There were statistically significant lower mean IKS scores when the femoral component was placed in valgus compared with varus (p \ 0.001) or neutral (p = 0.002) positions. | |  |  |  |
| Matziolis et al 2010 | KSS  WOMAC  SF36 | | No | 50 | Absolute malpositioning of the femoral component:  Group A (25 participants): 4.2°±1.4°  Group B (25 participants): 40.1°±1.4° | No significant difference in any of the PROMs scores assessed between the two groups. | |  |  |  |
| Gothesen et al 2014 | **KSS** | | **No** | **175** | No data provided | No significant difference in KSS scores assessed between the two groups. | |  |  |  |
| Czurda et al 2010 | **WOMAC**  **KSS** | | **No** | **38** | No data provided | No significant difference in PROMs scores between the two groups | |  |  |  |
| Nicol et al 2010 | **KSS** | | **No** | **45** | 6 outliers classed as >6±3° valgus | No significant difference between malalignment in this parameter and KSS score | |  |  |  |
| Longstaff et al 2009 | KSS | | Yes | **146** | 133 knees classed as good (±° of neutral). 13 knees classed as bad (outside the ‘good’ parameters) | The patients with a good coronal femoral alignment had a significantly better functional outcome at 1 year compared to the badly aligned (P =.013) | |  |  |  |

cFA- Coronal femoral angle, cTA – Coronal tibial angle, **, KSS – Knee society score, WOMAC - Western Ontario and McMaster Universities Arthritis Index, OKS – Oxford knee score, SF-36 – Short form – 36.**

| Table 3-s: sTA, sFA -Studies investigating the association between implants’ sagittal malalignment and Patient reported outcome measures | | | | | |
| --- | --- | --- | --- | --- | --- |
| Author | Outcome Measure | Association between malalignment and worse outcome | Sample size | Alignment data | Findings |
| Bankes et al 2003 | **KSS** | **No** | **198** | **Sagittal alignment of femoral component:** The mean sagittal femoral angle of the 198 participants was 4.05°+/- 1.21° (SD) | There was no significant difference between knees in acceptable and suboptimal alignment in terms post operative knee function scores. |
|  |  |  |  | **Sagittal alignment of tibial component:** The mean sagittal tibial angle of the 198 participants was 89.67°+/- 1.96° (SD) |  |
| Stulberg et al 2008 | **KSS** | **No** | **52** | **Sagittal alignment of femoral component:** The mean sagittal femoral angle of the 52 patients was 1.73° | There was no significant difference between malalignment in the sagittal parameter and KSS scores |
|  |  |  |  | **Sagittal alignment of tibial component:** The mean sagittal tibial angle of the 52 patients was -2.93° of valgus |  |
| Longstaff et al 2009 | KSS | No | **146** | **Sagittal alignment of femoral component:** 90 knees classed as good (±2° of neutral). 56 knees classed as bad (outside the ‘good’ parameters) | Patients with a good sagittal femoral and tibial alignment demonstrated a trend to better function at 1 year. However this relationship was non-significant |
|  |  |  |  | **Sagittal alignment of tibial component:** 95 knees classed as good (+1 - +5° of neutral). 51 knees classed as bad (outside the ‘good’ parameters) |  |
| Bach et al 2009 | KSS, HSS, Bristol score, Nottingham health profile | No | **98** | **Sagittal alignment of femoral component**: 47.9% of participants within normal range. Mean femoral component angle was 4.6° of flexion. | No significant correlation was found between the prosthetic alignment (sagittal femoral and tibial angle) and the mean clinical score outcome (p>0.05). |
|  |  |  |  | **Sagittal alignment of tibial component** 52% of participants were within normal range with a mean tibial component angle of 86.3° of flexion |  |

**sTA - Sagittal tibial angle, sFA – Sagittal femoral angle, KSS – Knee society score, HSS - Hospital for special surgery score**

| Table 4-s: Table summarising the results of the studies demonstrating an association between alignment in the axial parameter and patient reported outcome measures. | | | | | |
| --- | --- | --- | --- | --- | --- |
| Author | Outcome Measure | Association between malalignment and worse outcome | Sample size | Alignment data | Findings |
| Barrack et al 2001 | KSS | Yes | 28 | Control group: 2.6° external rotation. Malaligned group the mean was 4.7° internal rotation | Tibial component rotation and combined component rotation were correlated with lower KSS (clinical) and the presence of anterior knee pain. |
| Bell et al 2012 | OKS  VAS | Yes | 112 | Tibial component rotation: Control group defined as 5.8° ER [16.8° IR to 15.8° ER] vs cases group 3.4° IR [19.6° IR to 18.0° ER] | There was a signiﬁcant difference between the two cohorts with increased numbers of patients in the painful cohort with excessively internally rotated tibial (p=0.0003) and femoral (p=0.014) components and with internally rotated combined component (p=0.0003) and mismatched component rotations (p=0.0001). |
|  |  |  |  | Femoral component rotation: Control group defined as 3.9° ER [7.9° IR to 9.3° ER] vs Cases group: 2.3° IR [19.5° IR to 6.3° ER] |  |
|  |  |  |  | Combined rotational angle: Control group defined as 8.7° ER [16.1° IR to 23.7° ER] vs Cases group 7.1° IR [27.2° IR to 13.6° ER] |  |
| Rienmüller et al 2012 | KSS | No | 204 | Femoral component rotational angle: In 96 knees were within the range 0 ± 3° (neutral rotation group). 108 were in the outlier group. | No statistically significant difference could be seen in relation to KSS (knee score [KS] and function score [FS]) or range of motion |
| Howell et al 2013 | OKS  WOMAC | No | 101 | Rotation of the tibial component in relation to the femoral component: 98 knees within 0±10° and 2 knees classed as outliers >±10° | No significant difference in WOMAC and OKS scores between the groups |
| Lutzner et al 2010 | KSS | Yes | 73 | 9 participants had a rotational mismatch of >±10°. | Significantly worse knee society functional scores within the group who had rotational mismatch of >±10° (p=0.001), there was also a significant difference in overall KSS scores between the groups (p=0.04) |
| Czurda et al 2010 | WOMAC  KSS | Yes | 38 | No data provided | Patients with rotational malalignment had a sevenfold higher probability of suffering from post-operative pain (P = 0.033). |
| Nicol et al 2010 | **KSS** | **No** | **45** | Combined rotation; Painful Mean 8.0 IR Range (25.6 IR- 22.1ER) vs Painfree Mean 1.3 ER Range: (10.7 IR- 14.3 ER) (p0.001)  Mismatch rotation; Painfull Mean 2.6 IR Range (25.6 IR- 21.1 ER) vs Painfree 3.1 ER Range (10.3 IR - 22.1ER) (p0.025). | In the painful group there was more cases with femoral internal rotation over 6 degrees and tibial internal rotation of 9 degrees. (p<0.001) |
| Longstaff et al 2009 | KSS | No | **146** | 68 participants had a ‘good’ combined rotational angle (+/- 2° of neutral). There were 78 participants classed as outliers (+/-3°), (>5°) | Patients with a good rotational femoral and tibial alignment demonstrated a trend to better function at 1 year. However this relationship was non-significant |

**IR - Internal rotation, ER - External rotation, KSS – Knee society score, WOMAC - Western Ontario and McMaster Universities Arthritis Index, OKS – Oxford knee score, VAS - Visual analogue score for pain.**


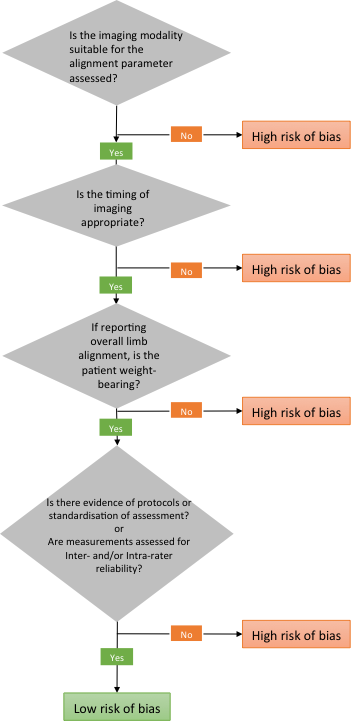


**Figure 1-s: Radiological Assessment Quality (RAQ) criteria for assessing alignment.** The evaluation was done using a five yes/no question checklist that was devised for this review. A sensitivity analysis was performed to determine if the quality of the radiological methods was an important factor in the outcome. The rationale for each set of questions was as follows: **The suitability of the imaging modality used:** Overall limb alignment is better assessed on a whole leg radiograph compared to a short film radiographs^[35]^ and Short film x-rays are used for the assessment of component’s anatomical alignment^[36]^. **The timing of the imaging:** Malalignment on images acquired several years following surgery may be secondary to implant subsidence/migration^[37]^. **The patient’s weight bearing status at the time of imaging:** the relationship between the bony and soft tissue parts of the knee joint is most visible during stressing manoeuvre such as weight bearing^[38]^. **Indication of standardisation when acquiring the images:** Non-standardised protocols for acquiring images can result in inconsistent magnification and rotation, introducing a source of bias^[39,49]^. **Evidence of rater reliability when assessing the images for alignment:** To ensure consistency^[39,40]^.
